# Emergence of Multidrug-resistant *Salmonella* Paratyphi B dT^+^, Canada

**DOI:** 10.3201/eid1007.030862

**Published:** 2004-07

**Authors:** Michael R. Mulvey, David Boyd, Axel Cloeckaert, Rafiq Ahmed, Lai-King Ng, the Provincial Public Health Laboratories

**Affiliations:** * Health Canada, Winnipeg, Manitoba, Canada;; † Institut National de la Recherche Agronomique, Nouzilly, France; 1 Provincial Public Health Laboratory Members: Lewis Abbott (Prince Edward Island), Kevin Forward (Nova Scotia), Glenna Hardy (New Brunswick), Greg Horsman (Saskatchewan), Judy Isaac-Renton (British Columbia), Frances Jamieson (Ontario), Jean Joly (Quebec), Jutta K. Preiksaitis (Alberta), Sam Ratnam (Newfoundland), Paul Van Caeseele (Manitoba).

**Keywords:** Multidrug resistant, *Salmonella* Paratyphi B, *Salmonella* genomic island, *Salmonella enterica* serovar Paratyphi B dT^+^

## Abstract

We document an increase in the number of multidrug-resistant *Salmonella enterica* serovar Paratyphi B dT^+^ identified in Canada. Most of these strains harbor *Salmonella* genomic island 1 (SGI1). Further studies are needed to determine factors contributing to the observed emergence of this multidrug-resistant strain.

*Salmonella* genomic island 1 (SGI1) was first characterized in *Salmonella enterica* serovar Typhimurium definitive phage type 104 (DT104). It consists of a 43-kb DNA segment harboring genes responsible for the pentaresistance phenotype ampicillin, chloramphenicol, streptomycin, sulfonamide, and tetracycline (ACSSuT) and is inserted into the chromosome at the end of the *thdF* gene (1). Complete nucleotide sequence of this region revealed 44 open reading frames, of which some displayed homology to genes associated with plasmid transfer, which suggests SGI1 may be at least partially plasmidic in origin (2). The SGI1 is associated with the multidrug-resistant DT104 clone that has disseminated worldwide (3). Recently, a number of reports have described SGI1 and variants in other *Salmonella* serovars, including *S*. Agona, *S*. Albany, and *S*. Paratyphi B dT^+^ (2,4–6). The worldwide dissemination of the DT104 clone has led some investigators to suggest SGI1 contains genes that may provide a selective advantage to the organism (2,4). We document the rapid increase of *S*. Paratyphi B dT^+^ isolates harboring SGI1 in Canada and provide further evidence to support that other unknown genetic factors may contribute to the rapid dissemination of multidrug-resistant strains of *Salmonella* serotypes globally.

## The Study

**Figure 1:**
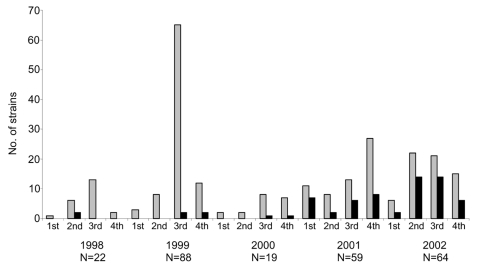
Total number of *Salmonella enterica* serovar Paratyphi B dT^+^ identified in Canada (gray bars) and the number of multidrug-resistant *S*. Paratyphi B dT^+^ identified over the same period (black bars), by quarter.

This report is a result of a collaborative effort between the National Microbiology Laboratory (NML), Health Canada, and the Provincial Public Health Laboratories (PPHLs) in Canada. The PPHLs represent every province in Canada and also include the Yukon and North West Territories. All *S*. Paratyphi B dT^+^ identified at the PPHLs were forwarded to the NML for additional characterization. Between 1998 and 2002, 252 *S*. Paratyphi B dT^+^ strains were submitted to the NML, of which 246 were from a human source. Distribution of the strains over time is shown in Figure 1. Incidence of *S*. Paratyphi B dT^+^ has generally increased since 1998; the spike in *S*. Paratyphi B dT^+^ in the third and fourth quarters of 1999 can be attributed to an outbreak in British Columbia, Alberta, and Saskatchewan caused by contaminated alfalfa sprouts (7). In addition to this large outbreak, additional outbreaks were reported between 1998 and 2002; however, each outbreak was small, and most involved fewer than six persons.

Antimicrobial susceptibility testing was performed on all strains by using the disk-diffusion method as described by the National Committee for Clinical Laboratory Standards (8). Susceptibilities were determined for ampicillin (A), chloramphenicol (C), ciprofloxacin (Cp), streptomycin (S), sulfamethoxazole (Su), tetracycline (T), and trimethoprim (Tm). Of the 237 strains examined for susceptibility, 123 (52%) were susceptible to all antimicrobial agents tested. Sixty-seven strains (28%) displayed the pentaresistance phenotype (ACSSuT), and the second most prevalent resistance profile was Su (n = 41, 17%). Single strains displayed the phenotypes A, T, CSSu, ASuTm, ASSu, and ACSuTTm. A significant increase was observed in ACSSuT strains over the time period from 1998 to 2002 (p < 0.001). Rates for the years are as follows: 1998, n = 2 (2%); 1999, n = 4 (18%); 2000, n = 2 (10.5%); 2001, n = 23 (39%); and 2002, n = 36 (58%). No large outbreaks were reported during this time period. To determine if the pentaresistance phenotype was caused by SGI1, polymerase chain reaction (PCR) was used to detect integrons and the left (*thdF*-S001) and right (S044-*yidY*) junctions of SGI1 as previously described (2). Of the 67 strains identified with the ACSSuT phenotype, 63 contained 1.0-kb and 1.2-kb integrons and left and right junctions of SGI1, which suggests that these strains contained SGI1 inserted into the same location on the chromosome as was described for DT104 (1). One strain with the ACSSuT phenotype contained 1.0-kb and 1.2-kb integrons and gave a PCR product for the left junction amplification reaction but not the right junction, which suggests that a portion of SGI1 downstream of the integrons was missing. Three strains with the ACSSuT phenotype did not give the characteristic size products for integron (0.7 kb, n = 2; 2.0 kb, n = 1), and all were negative for the junction PCR, which suggests that these strains most likely harbored other resistance genes giving the ACSSuT phenotype. Only four strains with the ACSSuT phenotype were identified from a nonhuman source (one poultry, three environmental). Although the three environmental isolates contained SGI1, the ACSSuT strain isolated from poultry did not; instead, it contained a 2-kb integron. Of the human isolates for which source was reported for the ACSSuT strains (n = 53), all were isolated from stool, with the exception of three that were isolated from blood. To ensure the SGI1 was intact, a selected number of isolates were subjected to additional PCR with primers representing all regions of the 44-kb SGI1 element (Table). PCR conditions used were previously described (2). DNA from all of these strains gave positive reactions for all primer sets described, which suggests SGI1 was intact in these strains.

**Table:** PCR primer to detect various regions of SGI1

Set	Primer	Primer sequence (5´ to 3´)	Coordinates^a^	Product size (bp)
St1	U9-L1 P1-R1	TACTACAAGCAGATAACGCC TAGAAACGACAAAGCGCGTG	2771–2790 3660–3679	909
St3	P134-L1 P134-R2	AATCGACACGCGCTGTATTG CTTCCCATAATGCCGCAATG	16350–16369 17287–17306	957
St4	P134-L1 P134-R1	TGACCCAATTCCAAAGCCAC GTGTTTGGGCAAGATCCCAG	16784–16803 17820–17839	1490
St5	St2-GP21 St2-GP6	ATAACGGCAGGTTCCGGTTC CGATGAAGCGCACAAATTTG	20173–20192 21089–21108	936
St6	St2-GP24 St2-GP28	TCAAGATTCCTATCTGCAGG AGAGTTACTAGACCAAGCGC	24363–24382 25182–25201	838

^a^Coordinates from accession no. AF261825.

**Figure 2:**
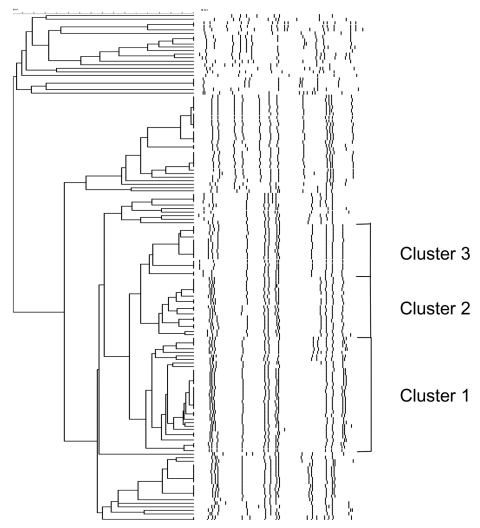
Dendrogram depicting the DNA fingerprints of *Salmonella enterica* serovar Paratyphi B dT^+^ identified from 2000 through 2002. Multidrug-resistant clonal groups labeled clusters 1 to 3 are shown.

*S*. Paratyphi B dT^+^ recovered from 2000 to 2002 were subtyped by using pulsed-field gel electrophoresis (PFGE) after DNA extraction and digestion with BlnI according to the standardized Salmonella protocol (9). PFGE-generated DNA profiles were entered into the BioNumerics software program version 3.0 (Applied Maths, St. Martens-Latem, Belgium) for analysis. Cluster analysis was performed by the unweighted pair-group method with arithmetic averages, and DNA relatedness was calculated on the basis of the Dice coefficient. In addition, all PFGE patterns were visually compared and assigned a number or letter identification (10). A dendrogram depicting the *S*. Paratyphi B dT^+^ BlnI macrorestriction patterns is shown in Figure 2. Of the 139 strains available to subtype (total = 142), visual comparison of the fingerprints revealed a total of 63 unique fingerprint patterns that grouped into 24 clusters. Analysis of the dendrogram revealed that all but three strains with the ACSSuT phenotype were grouped into three closely related clusters named clusters 1 to 3 (Figure 2). The three strains that did not cluster with the other ACSSuT strains did not harbor SGI1 as described above.

Cluster 1 contained 32 strains that represented 10 subtypes. Cluster 2 contained 17 strains that represented 7 subtypes, all of which showed <7 band differences between strains from cluster 1. Cluster 3 contained 15 strains that represented 6 subtypes, all of which showed fewer than seven band differences between subtypes in cluster 2, but had more than seven band differences between the cluster 1 fingerprints. In addition, six other strains identified before 2000 were subtyped. Five were other ACSSuT identified in 1998 (n = 1) and 1999 (n = 4), and all were identified in cluster 2. In addition, one *S*. Paratyphi B dT^+^ recently shown to harbor SGI1 that was isolated in Singapore from a fish was grouped into cluster 3 (4). The Canadian isolates in clusters 1 to 3 have been identified from multiple provinces, including British Columbia, Alberta, Manitoba, Ontario, and Québec, representing western and central regions of Canada.

## Conclusions

The emergence of multidrug-resistant *S*. Paratyphi B dT^+^ was documented recently in the Netherlands and Scotland (11,12). Some isolates had the ACSSuT susceptibility pattern and did not harbor any plasmids, which suggests the resistance is of chromosomal origin (12). However, in neither study was the presence of SGI1 or other resistance genes examined. We demonstrate the rapid emergence of multidrug-resistant *S*. Paratyphi B dT^+^ in Canada. The increase is due to three clusters, all of which contain the multidrug-resistant genomic island SGI1. That three closely related clonal groups were present suggests SGI1 may have inserted into the genome of *S*. Paratyphi B dT^+^ in three separate events, as shown by clusters 1, 2, and 3, or the insertion may have occurred once, with strains diverging over time. We also identified three strains with the ACSSuT phenotype that did not contain SGI1 sequences, which emphasizes the need to monitor genotypic resistance factors and not just phenotypic resistance traits to understand the dissemination of antimicrobial resistance.

The emergence of multidrug-resistant enteric pathogens is a concern because of the lack of suitable antimicrobial agents available to treat invasive infections. One organism that emerged in the 1990s is multidrug-resistant *S*. Typhimurium DT104, which harbors SGI1 (3). Along with the multidrug-resistant phenotype, reports suggest the strain may be more virulent than other salmonellae (13,14). However, in vitro studies have not shown any increase in invasiveness or survival in mammalian cells (15,16). Whether multidrug-resistant DT104 is more virulent remains to be determined, the underlying question remains: why has this clone of DT104 emerged as a major pathogen? Selective pressure, resulting from the widespread use of antimicrobial drugs in animals for growth promotion or prophylaxis, may have played a role in disseminating this organism. However, this factor may not completely account for its prevalence, because other multidrug-resistant strains of DT104 have emerged but have not disseminated internationally. Other factors may contribute to the international dissemination of this clone. For example, additional determinants in SGI1 may contribute to the fitness or virulence of *Salmonella* strains harboring it. In the present study, three closely related clusters of *S*. Paratyphi B dT^+^ carrying SGI1 have emerged in Canada and make up 46% (64 of 139) of all strains identified in 2001 and 2002. Furthermore, three additional *S*. Paratyphi B dT^+^ strains with the ACSSuT phenotype were identified that do not harbor SGI1 and do not appear to be rapidly increasing in Canada. In this hypothesis, SGI1 may predict the next emerging *Salmonella* serotype. Other factors, such as processing food products and the structure of the food distribution system, could play a role in disseminating these organisms. We continue to monitor this pathogen and are designing studies to improve understanding of the epidemiology of *S*. Paratyphi B dT^+^ in Canada. We suggest all strains of *Salmonella* with the ACSSuT phenotype be examined for SGI1.
